# Improved Moth-Inspired Algorithm Based on Fuzzy Controller

**DOI:** 10.3390/s25247633

**Published:** 2025-12-16

**Authors:** Zhoujing Lv, Dongxu Liu, Yu Wu, Huichao Zhu

**Affiliations:** 1 School of Control Science and Engineering, Dalian University of Technology, Dalian 116024, China; 2 School of Biomedical Engineering, Dalian University of Technology, Dalian 116024, China; 3 CRRC Information Technology Co., Ltd., Beijing 100041, China; 4 Key Laboratory of Intelligent Control and Optimization for Industrial Equipment of the Ministry of Education, Dalian University of Technology, Dalian 116024, China

**Keywords:** odor source localization (OSL), bio-inspired, fuzzy controller

## Abstract

In recent years, the demand for mobile robots to perform odor source localization tasks in dangerous environments has been increasing, and this technology has gradually become a focus of research. However, existing bio-inspired algorithms still have many limitations in real applications. Therefore, we attempt to design a moth-inspired algorithm integrated with a fuzzy control mechanism to enhance the robot’s ability to track odor sources in environments with dense obstacles. The goal is to improve the accuracy and efficiency of localization. Through multiple sets of simulation and real environment experiments, we observed that this algorithm achieved significant improvements in multiple indicators, including task success rate, search efficiency, and path planning quality. Compared with the traditional moth algorithm, its stability and adaptability in complex scenarios are also outstanding.

## 1. Introduction

Modern industrial development is advancing rapidly, and various chemical gases are increasingly utilized in industrial production [1] and daily life [2]. However, the rising frequency of their use also introduces significant safety hazards. Gas leaks not only lead to severe environmental pollution but also directly threaten human health. Thus, efficiently and accurately detecting and localizing leakage sources for timely remediation has become a critical issue. Compared with traditional manual detection methods, robot-based odor source localization (OSL) approaches founded on gas sensor technology [3] have demonstrated immense potential in both theoretical studies and practical applications owing to their advantages in continuous monitoring, enhanced safety, and real-time response [4,5,6].

Research on robot odor source localization technology can be traced back to the 1990s and has always been a hot topic in both domestic and international academic circles and engineering practices. In the early days, it mainly relied on a single gas sensor to locate through concentration gradients [7]. Although this method was simple to implement, it was prone to misjudgment in medium- to high-intensity turbulent environments. In moderate-to-high intensity turbulent environments, a single gas sensor is prone to misjudgment because turbulence leads to highly intermittent odor plumes, drastic concentration fluctuations, and non-monotonic gradients, rendering simple concentration gradient ascent strategies ineffective. Fundamental signal characteristics include time-domain features such as concentration amplitude, frequency, and duration. For non-turbulent environments, localization can be directly achieved by relying on stable concentration gradients. Conversely, in turbulent environments, it becomes necessary to analyze the statistical properties and intermittency of odor signals, and to employ multi-sensor arrays or active sampling strategies to overcome the inherent unreliability of instantaneous gradients. In recent years, with the advancement of sensor technology, artificial intelligence, computer vision, and swarm intelligence [8], modern odor source localization methods can be roughly divided into gradient algorithms [9], bio-inspired algorithms [10], probabilistic algorithms [11], and machine learning algorithms [12]. Among them, reinforcement learning, deep learning, and probabilistic algorithms, although having high accuracy, often require a large amount of data support and computing resources. Meanwhile, biomimetic algorithms, drawing inspiration from animal odor localization strategies, are characterized by their lower computational overhead, their potential for effective localization in resource-constrained environments, and their robust performance in complex, dynamic environments, offering a new research direction for odor source localization.

The odor localization strategies employed by animals have been refined over long periods of natural selection, and algorithms inspired by these high-performance biological systems exhibit tremendous potential [5]. Among them, the moth-inspired algorithm—derived from the mating behavior of male moths—has been widely applied in robot odor source localization due to its simplicity of implementation and superior localization performance.

The earliest applications of moth-inspired algorithms to robotic gas source localization can be traced back to the last century. Belanger et al. [13] developed a bio-inspired search algorithm by mimicking the behavior of moths tracking odor sources, conducting simulation tests in the virtual environment Digiduca. They found that a simple reflex model yielded a low success rate, but performance significantly improved upon introducing the moth’s characteristic periodic upwind turning mechanism. Subsequently, by employing genetic algorithms to optimize parameters, the model successfully identified two distinct efficient strategies: fast oscillation and slow oscillation. These behaviors corresponded, respectively, to the “upwind surge” when moths approach an odor source and the “casting search” when they lose the odor plume.

Early research on the Moth-Flame Optimization Algorithm focused on the module design that mimics the behavior of moths. Ferri et al. [14] were inspired by the behavior of male moths locating their female partners through olfaction and proposed a spiral algorithm. If the robot detects that the gas concentration peak is higher than the initial threshold, it considers that its current position is closer to the gas source compared to its previous position, and then it starts the next round of spiral movement; otherwise, it continues the current spiral movement.

Building directly on biological observations of male moth navigation, Lochmatter et al. [15] devised a mapless navigation framework. This algorithm emulates the following biological process: when a female moth releases a mixture of pheromones, these chemicals are carried by the wind to form a distinct plume structure; upon detecting this plume, the male moth initially flies upwind—a behavior termed “surge”—to track the pheromone molecules. Pheromone plumes in varying turbulence frequently disrupt the moth’s signal tracking during surging. To remedy this, it adopts an orthogonal search strategy (termed “cast”) to reacquire the pheromone signal. This behavior gives rise to what is known as the surge-cast algorithm; if the cast phase is replaced with spiral motion, the algorithm is referred to as the surge-spiral algorithm.

Subsequently, Waphare et al. [16] proposed an improved strategy based on both the surge-cast and surge-spiral algorithms. In their method, a gas sensor is installed on each side of the robot, and by comparing the readings from the left and right sensors, the initial search direction (i.e., whether to employ casting or spiral maneuvers) is determined. Their experimental results have shown that this improvement leads to a significant enhancement in overall performance.

Evidently, spiral algorithms rely on instantaneous values continuously obtained by sensors under concentration gradients, and similarly, dual-sensor surge-cast methods also leverage this type of gradient information to enhance moth-inspired approaches. However, in dynamic and often turbulent environments, concentration gradients prove to be highly unreliable. This unreliability stems not merely from airflow variability, but rather from the severe fluctuations in turbulent velocity leading to local instantaneous concentrations exhibiting complex physical characteristics such as high intermittency, drastic fluctuations, and non-monotonic gradients. These phenomena, extensively elucidated by Jiang et al. [17], render navigation strategies reliant on instantaneous concentration gradients exceedingly challenging, and even prone to misjudgment.

Against this backdrop, the introduction of fuzzy inference methods [18] has emerged as an effective solution. Wang et al. designed a fuzzy controller that allows the robot to continuously perceive its environment (e.g., odor concentration, rate of concentration change, and even potential wind direction inferred from multi-sensor or historical data) and dynamically adjust its trajectory parameters based on its current search state. Utilizing fuzzy inference, this algorithm intelligently integrates multi-source environmental information. Upon detecting an odor signal, it effectively determines the odor source direction and adjusts its movement strategy to proceed upwind.

Studies have shown that when the airflow environment exhibits turbulent characteristics, the robot expands its search trajectory, covering a larger area for extensive exploration. Conversely, as it approaches the odor source, its search trajectory becomes confined to a smaller region.

Despite the advancements achieved by moth-inspired algorithms in odor source localization, existing computational models and their implementations continue to face several challenges in practical applications. These challenges often stem from an oversimplification of the intricate biological mechanisms involved:

(1) Robot locomotion in complex, obstacle-rich environments is susceptible to interference, leading to fragmented or discontinuous search paths and compromising their completeness.
(2) During the initial search phase, current algorithms frequently employ random directional initialization. This contrasts with natural moth strategies, which leverage mechanisms like airflow direction sensing and bilateral olfactory comparison for efficient initial plume detection and localization.
(3) Odor source identification strategies are oversimplified, typically relying solely on whether the current concentration surpasses a predefined threshold. This approach fails to fully exploit the richer sensory information utilized by biological systems, such as dynamic changes in odor intensity, temporal frequency of encounters, and spatial distribution patterns.
(4) The use of fixed parameter settings in algorithms hinders their ability to adapt to the inherent dynamic nature of odor environments. This rigidity contrasts sharply with the remarkable adaptive capacity of biological organisms, which continuously adjust their behavior and parameters in real-time based on environmental feedback.

To address these issues, this paper proposes a bioinspired algorithm based on a fuzzy controller, aiming to achieve precise and rapid odor source localization by mobile robots in complex, obstacle-laden environments. Specifically, the proposed approach:

(1) Employs a rebound strategy to guide the robot in obstacle avoidance;
(2) Promptly records the robot’s movement direction upon exiting the plume and selects the opposite side for the subsequent surge or initial cast based on the detected concentration;
(3) Optimizes the odor source identification strategy by analyzing the spatial clustering of regions where the concentration exceeds a preset threshold;
(4) Integrates the fuzzy inference method with the traditional moth-inspired algorithm to realize dynamic regulation of both parameters and behavior.

The structure of this article is arranged as follows: The second part elaborately introduces the three-stage improvement methods of the traditional moth-like algorithm and discusses the adaptive parameter and behavior adjustment mechanism based on the fuzzy controller. The third part presents the simulation and actual experimental results and conducts an in-depth analysis. Finally, the Conclusions (Section 4) summarizes the main content of this paper and proposes the direction for future research.

## 2. Methods Improvement

The traditional moth-inspired algorithm can be divided into three stages: plume search, plume tracking and odor source declaration. This section proposes corresponding optimization plans for the problems existing in each stage and elaborates them in detail in the three sub-sections, respectively.

### 2.1. Obstacle Avoidance Strategy and Search Enhancement

In the plume search stage, the traditional moth-inspired algorithm often employs strategies for plume contact and re-contact, such as the Z-shaped method, to detect the odor plume in the surrounding environment. This method requires the robot to maintain a certain Angle with the wind direction when advancing, so as to form a Z-shaped movement path. This approach mirrors a consistent feature observed in many biological odor-tracking strategies. This movement mode simulates behavior extensively observed and first investigated in moths, and also shares similarities with strategies used by other insects, such as dung beetles when tracking the odor plume from cow dung. The specific process of this method is as follows:

(1) Linear travel stage: The robot starts from the initial position and travels a fixed distance along the preset direction. This advance distance can be flexibly adjusted based on practical conditions. In real-world applications, experts familiar with the environment often designate a target navigation point based on experience; however, if there is insufficient information regarding the approximate location of the odor source or the gas concentration distribution, this step may be omitted.
(2) Turning Phase: After completing a straight-line motion, if the robot has not detected an odor or met the designated target conditions, it then alternates its turns at a preset angle while maintaining a specific angle relative to the wind direction. By continuously repeating this turning process, the overall motion trajectory forms a “Z” pattern.

However, the forward direction in this method is essentially predetermined, rendering it difficult to ascertain an accurate initial heading in environments lacking sufficient information, whereas in information-rich settings it signifies excessive human intervention [19]. Moreover, when obstacles are encountered, the robot’s motion is easily disrupted, leading to a loss of trajectory continuity and integrity.

In light of these limitations, this section proposes an enhancement to the turning phase of the traditional Z-shaped method by introducing a rebound behavior strategy. Pyk et al. [20] previously employed a vision model based on locust LGMD neurons, fusing visual modalities to achieve predictive obstacle avoidance. While effective in controlled environments, this approach exhibited high reliance on sensor performance, ambient lighting conditions, and computational resources, and its behavior priority override mechanism could potentially interrupt the continuity of the search. In contrast, our proposed rebound behavior strategy no longer relies on complex visual modules, instead utilizing the robot’s inherent motion control and basic contact sensing, thereby achieving a lighter-weight and more easily deployable obstacle avoidance solution. This strategy is designed to enable the algorithm to perform more efficient search and obstacle avoidance in complex environments populated with obstacles. Specifically, when no obstacles are present, the robot maintains a fixed angle relative to the wind during its upwind search and performs alternating turns at the designated angles. However, upon encountering an obstacle, the robot deviates from its current trajectory by a predetermined angle so as to autonomously avoid the obstacle and systematically scan the search area.

More specifically, the robot initiates its search by proceeding in the upwind direction. In the absence of obstacles, the robot turns by an angle $\beta_{z}$ relative to the upwind direction; however, when an obstacle impedes its path, the robot adjusts by shifting its current heading by an offset angle $\psi_{z}$ to bypass and move away from the obstruction. It should be noted that the initial turn is by default toward the left; if, after turning left by $\psi_{z}$, an obstacle still lies directly ahead, the robot will then turn right by $2\psi_{z}$ (i.e., a right turn of $\psi_{z}$ relative to its initial heading). Should these adjustments fail to provide an unobstructed path, the robot reverses for a certain distance before reinitiating the Z-shaped search pattern. Experimental validation indicates that a reasonable range for the turning angle $\beta_{z}$ is between 30° and 60°, while the rotation angle $\psi_{z}$ should reasonably fall within 90° to 135°.

**Figure 1:**
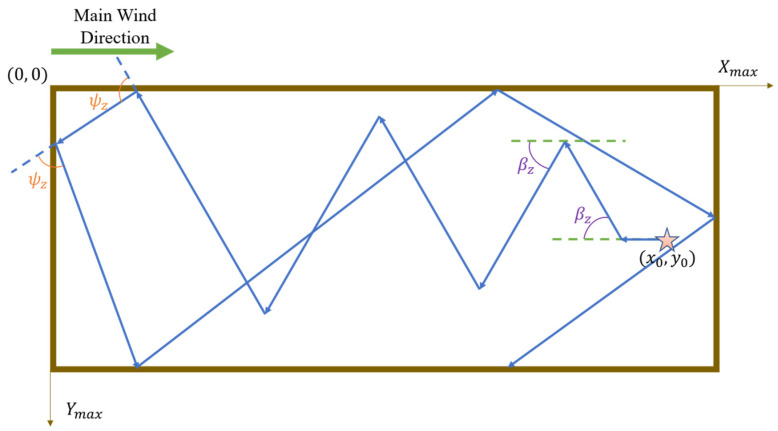
Robot performs bounce behavior to avoid walls. The five-pointed star represents the starting point, and the arrows indicate the robot’s movement path.

**Figure 2:**
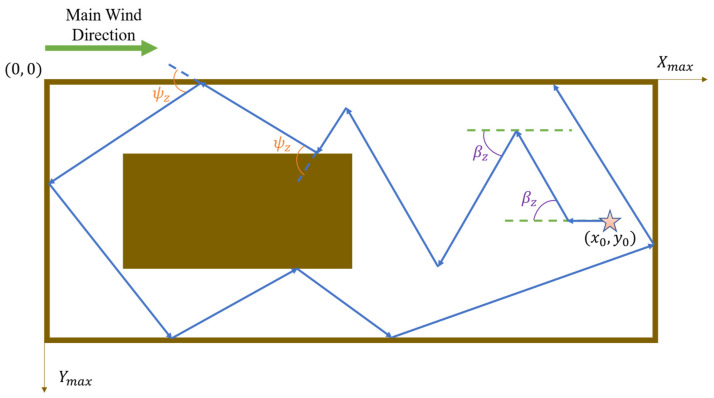
Robot performs bounce behavior to avoid obstacles and walls.The five-pointed star represents the starting point, and the arrows indicate the robot’s movement path.

The rebound behavior simulates the manner in which a billiard ball rebounds after striking the edge of a table, thereby enabling the robot to move in a direction that maximally distances it from obstacles. This obstacle avoidance behavior is illustrated in Figure 1 and Figure 2, where the origin of the experimental environment is designated as point (0, 0); $X_{max}$ and $Y_{max}$ represent the maximum distances along the *x*-axis and *y*-axis (i.e., the environmental boundaries); the robot’s initial position is denoted as $\left(x_{0},\;y_{0}\right)$; $\beta_{z}$ represents the heading angle during the Z-shaped search, and $\psi_{z}$ denotes the rotation angle employed for obstacle avoidance. It is important to note that the primary wind direction indicated in the figures is for demonstrative purposes only; in actual experiments, the wind direction is determined by data collected via the robot’s sensors.

The integration of the rebound obstacle avoidance strategy aims to address the limitations of the traditional zigzag odor plume search method in complex environments. It not only significantly enhances the applicability of this search method in obstacle-dense areas, ensuring the robot can systematically and efficiently explore the entire target search region, thereby accelerating the localization of the odor source. It is important to emphasize that, while this section primarily focuses on odor source search, the proposed rebound obstacle avoidance mechanism, as a general motion planning strategy, is equally effective for various robot navigation tasks that require autonomous obstacle avoidance. For instance, when performing search tasks, it ensures the continuity of the robot’s path, preventing interruptions caused by obstacles; and when executing point-to-point navigation tasks, it also provides efficient obstacle avoidance capabilities. This versatility makes the rebound obstacle avoidance strategy highly significant in improving robot autonomy and task efficiency, and it can be conveniently transplanted to other stages of bionic algorithms, thereby contributing significantly to enhancing the success rate and efficiency of various robot tasks.

### 2.2. Side Leaving the Plume Guided Search

The surge behavior is designed to ensure that once the robot detects the odor plume, it can efficiently track its trajectory. However, in traditional methods, as soon as the robot exits the plume, a cast behavior is immediately activated to force a rapid reentry [21]. Such frequent switching not only increases the overall algorithmic complexity but also tends to induce unnecessary path oscillations. Moreover, while some conventional approaches may incorporate past data to a limited extent, they often lack a mechanism for the systematic and adaptive integration of previously acquired information to dynamically guide the forward movement, which primarily remains determined by instantaneous wind direction. To address these issues, this paper introduces the concept of the Side Leaving the Plume (SLP), which identifies the lateral direction at which the robot departs from the plume. This strategy effectively reduces the frequency of behavior switching, simplifies the overall algorithm, and employs historical information to calculate the heading angle, thereby significantly increasing the probability of the robot remaining within the plume during subsequent tracking.

During the surge phase, the robot’s next heading angle, $\psi_{s}$, is computed using the formula:

(1) $$\psi_{s}=\phi +180^{{}^{\circ}}+SLP\cdot \beta_{s}$$

**Figure 3:**
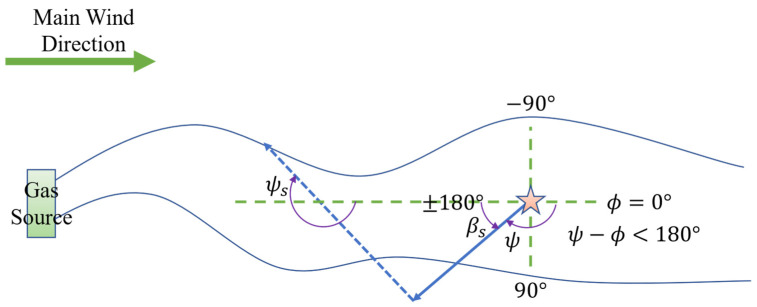
Robot determines SLP=1 to return to plume in surge behavior. The five-pointed star represents the robot’s starting position; the solid arrows indicate the robot’s initial path, while the dashed arrows represent the correction path taken to relocate the plume after leaving it.

**Figure 4:**
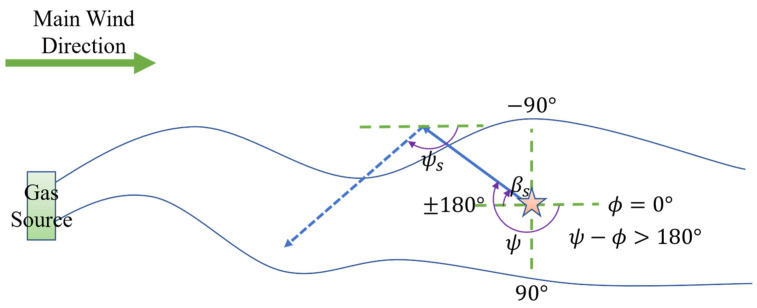
Robot determines SLP=−1 to return to plume in surge behavior. The five-pointed star represents the robot’s starting position; the solid arrows indicate the robot’s initial path, while the dashed arrows represent the correction path taken to relocate the plume after leaving it.

Here, ϕ represents the wind direction sensed at the robot’s current position, and SLP records the lateral departure from the plume. SLP is determined based on the difference between the robot’s current heading angle ψ and the wind direction ϕ (i.e., ψ−ϕ). As illustrated in Figure 3, if the robot departs the plume from its left side (i.e., $\left(\psi -\phi \right)<$ 180°), SLP is defined as 1; conversely, if it departs from the right side (i.e., $\left(\psi -\phi \right)>$ 180°), SLP is defined as −1, as shown in Figure 4. Here, both the length of the cast behavior and the trajectory parameters of the spiral search are adaptively determined by the fuzzy controller based on real-time environmental information, rather than being preset fixed values. This dynamic adjustment mechanism based on fuzzy logic significantly enhances the algorithm’s adaptability and robustness in complex and changing environments. In either case, the computed heading command directs the robot from the departure side back toward the plume, thereby enhancing the likelihood of reentry.

**Figure 5:**
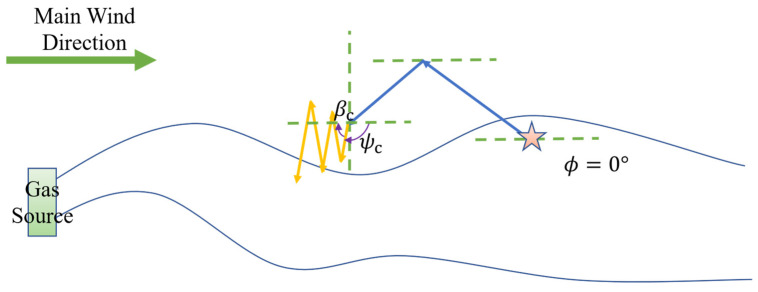
Robot attempts to re-enter the plume with SLP=1 by cast behavior. The five-pointed star indicates the robot’s initial position. The blue arrows represent the robot’s movement direction, and the yellow arrows indicate the movement direction determined by the behavior algorithm currently being executed by the robot.

**Figure 6:**
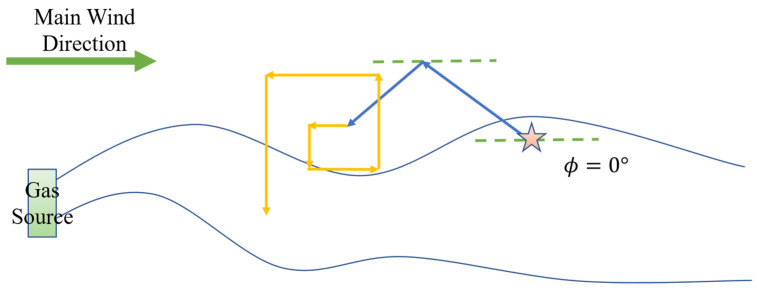
Robot attempts to re-enter the plume with SLP=1 by spiral behavior. The five-pointed star indicates the robot’s initial position. The blue arrows represent the robot’s movement direction, and the yellow arrows indicate the movement direction determined by the behavior algorithm currently being executed by the robot.

In the plume tracking process, the robot initially executes the “surge” behavior to approach the odor source; this represents the first stage of plume tracking in bioinspired algorithms. When the robot completely loses the plume signal, it immediately initiates a reverse surge behavior in an attempt to reenter the plume. Let t denote the current time, $t_{l}$ the time when the plume was last detected, and $t_{s}$ the maximum allowable duration for the surge behavior. If the robot remains outside the plume for a period such that ${t-t}_{l}>t_{s}$, it is forced to switch to cast or spiral behavior, marking the second stage of plume tracking. As depicted in Figure 5 and Figure 6, during the cast phase the robot’s next heading angle, $\psi_{c}$, is calculated by:

(2) $$\psi_{c}=\phi +180^{{}^{\circ}}-SLP\cdot \beta_{c}$$

In this formula, $\beta_{c}$ is the offset angle between the robot’s motion direction and the upwind direction, typically ranging from 75° to 90°. The role of SLP here remains the same as in the surge phase: to guide the robot from the direction of departure back into the plume, corresponding to the initial wind-perpendicular direction in the spiral phase. Essentially, both cast and spiral behaviors aim to bring the robot back into the plume; therefore, the traditional spiral algorithm is simplified to primarily consider the initial wind-perpendicular spiral direction, step length, and step-length increment.

Throughout the entire plume tracking process, a transition to the source declaration phase is triggered when the detected gas concentration surpasses a certain threshold. It is important to note that, addressing the limitation of fixed parameters raised in the Introduction, this threshold is not a preset constant but is adaptively determined in real-time by the fuzzy controller, as will be elaborated in Section 2.4. If, during the cast or spiral behavior, the robot detects the plume but the gas concentration remains below the threshold, it reverts to the surge phase to continue tracking the plume. Conversely, if after a surge duration of $t_{c}$ (A variable whose value is dynamically generated by a fuzzy controller. Its value is not fixed, but is jointly determined by the robot’s real-time perception of environmental turbulence intensity, gas concentration, and signal loss duration.) the robot still fails to detect the plume (i.e., ${t-t}_{l}>t_{c}$), it returns to the initial plume search phase by reactivating the Z-shaped search to perform a broad search for plume information in the environment.

This integrated approach, which utilizes the concept of lateral departure (SLP) in conjunction with dynamically computed heading angles, not only minimizes unnecessary transitions between behaviors but also significantly enhances the robot’s ability to effectively reengage with the odor plume.

### 2.3. Suprathreshold Positional Aggregation

For probabilistic algorithms, determining the location of the odor source is relatively straightforward because these methods typically preset convergence thresholds; once the corresponding conditions are met, the odor source can be confirmed [22]. In contrast, gradient-based and bioinspired algorithms face greater challenges in odor source declaration. Although these methods also preset a threshold for gas concentration, relying solely on whether the instantaneous concentration exceeds [23] that threshold is overly simplistic and lacks sufficient analytical depth. Such a method is prone to misjudgments, potentially leading the robot to localize a pseudo-odor source.

This paper introduces an odor source declaration method based on Suprathreshold Positional Aggregation (SPA), which aims to enhance the success of odor source identification in bioinspired algorithms. Building upon the shared principle of utilizing spatial distribution, the SPA method’s principal advantage lies in its tight integration with the fuzzy adaptive framework presented in this work. Unlike standalone approaches, SPA’s parameters (e.g., the number of points N_sand distance threshold D_s) are dynamically adjusted by the fuzzy controller, creating a cohesive system that synergistically enhances both the plume tracking and source declaration phases for superior overall performance in complex settings.

In odor source localization tasks, when a robot gradually moves away from the odor source, the detection points where the concentration exceeds the threshold tend to be relatively dispersed along the airflow direction. Conversely, as the robot approaches the odor source, these suprathreshold detection points become densely clustered in the downwind region of the source. This phenomenon arises from the combined effects of odor molecule diffusion and environmental wind fields, leading to a significant increase in the spatial gradient of the measured concentration near the source [24]. The SPA algorithm leverages this spatial aggregation characteristic and comprises the following core steps:

(1) Sorting Suprathreshold Detection Points by Airflow Direction: The robot collects a series of *N_S_* detection points, each recording a gas concentration exceeding the threshold *C_T_*, which is dynamically adjusted by the fuzzy controller introduced in Section 2.4. These points are then sorted according to the dominant wind direction. Specifically, whenever a concentration reading exceeds the threshold, the detection point is appended to a sequence, which is re-sorted based on the current predominant wind direction so that the detection point nearest the upwind side is positioned at the end of the sequence. This sorting reflects the dynamic trajectory of the robot as it progressively approaches the odor source, with the dominant wind direction obtained by averaging data from the onboard wind sensor.
(2) Evaluating the Aggregation Degree of Detection Points: The sorted *N_S_* detection points are then assessed for clustering using a deliberately simple and computationally efficient geometric heuristic. This process involves calculating the distances between consecutive points in the ordered sequence—an operation with linear complexity *N_S_*. A cluster is identified if all these distances are below the threshold *D_S_*. This lightweight check serves as a practical and efficient alternativeto the computationally intensive probability map maintenance or entropy minimization employed in other methods, directly addressing the criticism of complex models and making it highly suitable for real-time implementation.

By analyzing the distribution of odor concentration measurements at various positions, the SPA algorithm extracts richer environmental information, thereby allowing for a more accurate assessment of the relative positional relationship between the robot and the odor source. When the detection points in the downwind region exhibit a high degree of clustering, it generally signifies that the robot is in close proximity to the odor source. This method not only fully exploits the spatial distribution characteristics of odor concentration but also overcomes the shortcomings of traditional methods that rely solely on single instances of high concentration detection. Consequently, SPA significantly improves the reliability and accuracy of odor source declaration.

### 2.4. Design of Fuzzy Controller

Fuzzy Logic was first proposed by Zadeh in 1965, with the aim of overcoming the rigidity of traditional Boolean logic where “any statement is either true or false”. Compared with traditional binary logic, fuzzy logic allows variables to take any value between 0 and 1, and this continuity is more in line with human thinking habits. It introduces the concept of a degree of truth to measure the extent to which a given object belongs to a particular fuzzy set, mirroring the linguistic variables found in natural language [25]. The fuzziness of fuzzy logic by allowing expressing reality to build a more flexible logic framework. Fuzzy Inference is a reasoning technique developed on the basis of fuzzy logic. It can effectively simulate the dynamic behavior of complex and nonlinear systems, and its rules have good interpretability, making it very suitable for odor source location systems.

#### 2.4.1. Overall Framework of the Fuzzy Controller

The fuzzy controller utilizes linguistic descriptions and fuzzy rules. Firstly, it performs fuzzification on the environmental data collected by sensors, such as air flow velocity, wind direction changes, and odor concentration, etc., and uses them as fuzzy inputs to determine the current environmental state. Then, based on the pre-defined fuzzy rules, it obtains the corresponding fuzzy output. Finally, it defuzzifies it to obtain specific parameter values suitable for the current environment, thereby achieving real-time dynamic adjustment of the robot’s search parameters [18]. This method ensures that the robot can maintain high search efficiency and positioning accuracy when facing constantly changing airflows and complex environmental conditions.

In the context of robot odor source localization, the selection and tuning of search parameters are crucial for both the efficiency and the accuracy of the search process. Traditional bioinspired algorithms typically determine the parameters suited to a given environment—such as the turning angle $\beta_{z}$, step length *T_z_*, and its increment ${∆T}_{z}$ in the Z-shaped search phase; the turning angle $\beta_{s}$, maximum execution time *t_s_*, initial step length $T_{s}$, and step length increment ${∆T}_{s}$ in the surge phase; the turning angle $\beta_{c}$, maximum execution time $t_{c}$, initial step length $T_{c}$, and step length increment ${∆T}_{c}$ in the cast or spiral phase; as well as the threshold concentration $C_{T}$, the number of threshold detection points $N_{S}$, and the distance difference threshold $D_{S}$ during the odor source declaration phase—through multiple preliminary experiments conducted before the formal search commences. Under the joint effect of these parameters, not only the path and range of the robot’s search are determined, but they also deeply participate in the decision-making process of switching between different search behaviors. Once these parameters are set, they remain unchanged throughout the entire search process.

**Figure 7:**
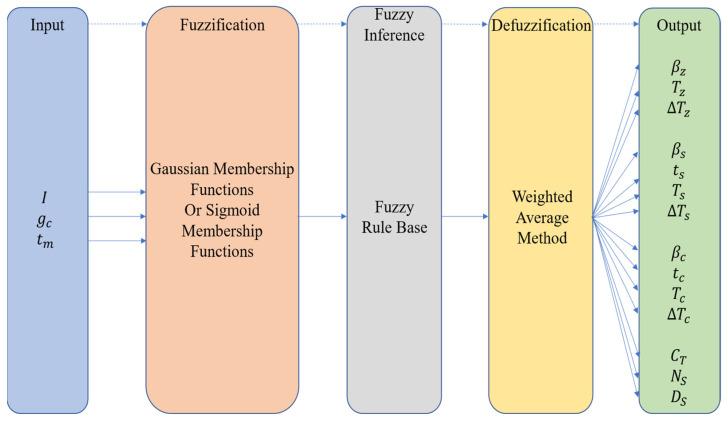
Fuzzy controller flowchart. The arrows indicate the direction of signal flow.

However, in actual environments, air flow conditions are constantly changing, and fixed parameters are difficult to adapt to complex and variable scenarios, thereby affecting the positioning effect. To solve this problem, this paper proposes a fuzzy controller based on environmental air flow information to make up for the deficiency of traditional bio-inspired algorithms in parameter setting, enabling the search parameters to be dynamically and adaptively adjusted according to real-time search conditions and environmental features. The specific process of this fuzzy controller is shown in Figure 7.

#### 2.4.2. Inputs and Outputs of the Fuzzy Controller

The fuzzy controller receives as inputs the turbulent intensity I present in the environment, the gas concentration $g_{c}$ measured at the robot’s current position, and the time interval $t_{m}$ during which the odor plume is not detected. Its outputs encompass several parameters across multiple search phases: in the Z-shaped search phase, the turning angle $\beta_{z}$, the initial step length $T_{z}$, and the step length increment ${∆T}_{z}$; in the surge phase, the turning angle $\beta_{s}$, the maximum execution time $t_{s}$, the initial step length $T_{s}$, and the step length increment ${∆T}_{s}$; in the cast or spiral phase, the turning angle $\beta_{c}$, the maximum execution time $t_{c}$, the initial step length $T_{c}$, and the step length increment ${∆T}_{c}$; and in the odor source declaration phase, the threshold concentration $C_{T}$, the number of threshold detection points $N_{S}$, and the distance difference threshold for these detection points $D_{S}$.

Parameter Initialization and Adaptive Adjustment: The parameters governed by the fuzzy controller are first initialized with baseline values derived from empirical studies and preliminary experiments, ensuring reasonable initial robot behavior. For instance, the turning angles ($\beta_{z},\beta_{s},\beta_{c}$) are typically initialized within ranges of 30–60°, 75–90°, and 75–90°, respectively, while step lengths ($T_{z},T_{s},T_{c}$) are set based on the scale of the experimental environment. The core innovation, however, lies not in these initial values but in their dynamic adaptation. The fuzzy controller continuously modulates these parameters in real-time based on the inputs (I,$g_{c}{,t}_{m}$). For example, a high turbulence intensity (I) will trigger an increase in the Z-search turning angle $\beta_{z}$ to promote exploration, while a high gas concentration ($g_{c}$) will cause a decrease in the surge step length $T_{s}$ to refine the search near the potential source. This mechanism replaces the static parameterization of traditional methods with a context-aware strategy, which is the primary contribution of this work.

Here, the turbulent intensity I is defined as the ratio of the standard deviation of the wind speed σ to the average wind speed $\bar{v}$; that is,

(3) $$I=\frac{\sigma }{\bar{v}}$$

In practical flow fields, turbulence is not only present in the primary wind direction but may also exhibit significant fluctuations in the lateral or vertical directions. However, most current wind speed and wind direction sensors are limited to two-dimensional measurements. Therefore, this paper computes the standard deviation σ and the average wind speed $\bar{v}$ using the wind speeds measured along the x- and y-axes on the horizontal plane.

(4) $$\sigma =\sqrt{\frac{1}{2}\left(\sigma_{x}^{2}+\sigma_{y}^{2}\right)}$$

(5) $$\bar{v}=\sqrt{v_{x}^{2}+v_{y}^{2}}$$

Considering the critical roles that airflow characteristics, odor concentration, and the robot’s current search state play in odor source localization within the experimental environment, we strive to comprehensively convey this essential information to the fuzzy controller. To achieve this, we first calculate the turbulent intensity I using data collected from wind speed and wind direction sensors. We then combine this value with the odor concentration $g_{c}$ measured by the gas sensor and the time interval $t_{m}$ during which the robot fails to detect an odor plume, thereby providing the fuzzy controller with a robust set of inputs for subsequent dynamic parameter adjustments.

#### 2.4.3. Fuzzification

**Figure 8:**
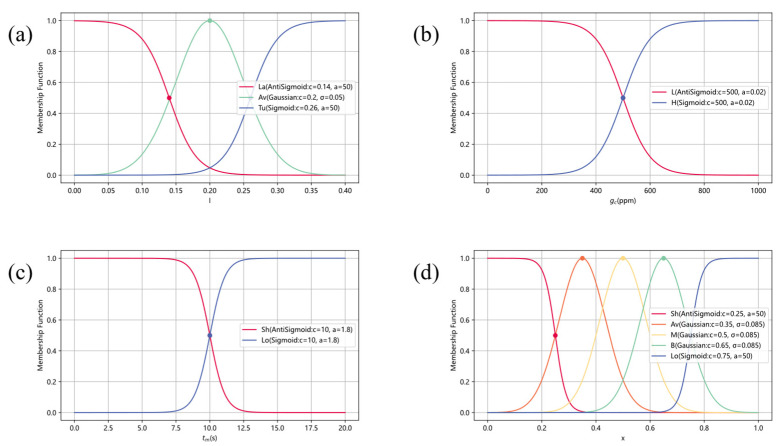
Inputs (**a**–**c**) and outputs (**d**) of the fuzzy controller and their corresponding membership functions.

The formation of fuzzy input is to map the collected precise input data to various pre-defined fuzzy sets through the fuzzification process. During the fuzzification process, membership functions with values ranging from 0 to 1 are used to quantify the degree of certainty that the input data belongs to a certain fuzzy set. Figure 8 shows the fuzzy input and fuzzy output and their corresponding membership functions. We choose the Gaussian membership function or the Sigmoid membership function to model all fuzzy sets. These two membership functions, with their smoothness and continuity, provide a tool that is convenient for mathematical analysis and parameter tuning for each fuzzy set. Among them, the formula of Gaussian membership function is as follows:

(6) $$\xi \left(x\right)=\exp\left(-\frac{{\left(x-c\right)}^{2}}{2\sigma^{2}}\right)$$

The formula for the Sigmoid membership function is as follows:

(7) $$\xi \left(x\right)=\frac{1}{1+\exp\left(-a\left(x-c\right)\right)}$$

It should be noted that the inputs to the fuzzy controller typically refer to the raw, precise data acquired by the robot or system, whereas the fuzzy inputs are obtained by subjecting these raw data to the process of fuzzification, which quantifies their degree of membership within various fuzzy sets. In other words, the raw input data are transformed via membership functions into fuzzy inputs that provide information in a format more conducive to subsequent fuzzy inference. Thus, although both types of information are essential to the fuzzy controller, the former remains as unprocessed, precise data, while the latter constitutes the processed form achieved through fuzzification.

#### 2.4.4. Fuzzy Inference

Fuzzy inference plays a critical role in the fuzzy controller by mapping fuzzy inputs to fuzzy outputs through a set of decision rules. To avoid an excessive number of rules and an overly complex control system, only a limited number of fuzzy sets are defined for each input variable. For instance, the turbulent intensity variable I is partitioned into three fuzzy sets—laminar (La), average (Av), and turbulent (Tu); the sensed gas concentration $g_{c}$ is classified into low (L) and high (H); and the time interval $t_{m}$ during which no odor plume is detected is divided into short (Sh) and long (Lo). Conversely, all output variables are normalized to the range [0, 1], and five fuzzy sets—very small (VS), small (S), medium (M), big (B), and very big (VB)—are uniformly assigned to quantify output levels from low to high. The fuzzy rules, which underpin the map between fuzzy inputs and outputs, integrate environmental wind field characteristics and odor detection information to regulate the output parameters and thereby formulate the robot’s response strategy under various search conditions; the design of these rules draws on extensive experience from previous bioinspired algorithm research.

Research indicates that when operating in turbulent conditions, a robot can significantly enhance search efficiency by increasing its crosswind displacement to cover a broader area [17]. In laminar conditions, while a direct upwind progression is generally effective, it is crucial to note that the plume narrows towards the source. Therefore, the strategy should dynamically shift: an initial emphasis on direct upwind movement is efficient for long-range approach, but upon detecting high concentration, the search must be refined (e.g., by reducing step size) to account for the narrowing plume and pinpoint the source accurately. Accordingly, the fuzzy controller incorporates distinct motion parameter adjustment strategies: an exploration strategy is adopted in turbulent environments to thoroughly search unknown areas for initial plume localization, while an exploitation strategy is implemented in laminar environments to conduct a meticulous local search using existing plume information. In addition, both gas concentration and the duration for which the robot fails to detect the odor plume critically influence parameter settings. Higher gas concentrations suggest a closer proximity to the odor source, necessitating a reduction in the algorithm’s step length for finer search resolution; in contrast, prolonged non-detection indicates that the robot is likely outside the plume, warranting a reduction in the maximum execution time to prompt a swift behavioral transition. Moreover, the combined effects of gas concentration and non-detection duration further adjust parameters: when a high concentration is detected immediately upon plume contact, indicating that the robot is near and within the plume, the threshold concentration $C_{T}$ and the number of detection points $N_{S}$ should be increased while the distance threshold $D_{S}$ is decreased to confine the search area for precise localization; conversely, when low concentration is accompanied by extended non-detection, the search area should be expanded by decreasing $C_{T}$ and $N_{S}$ and increasing $D_{S}$.

**Figure 9:**
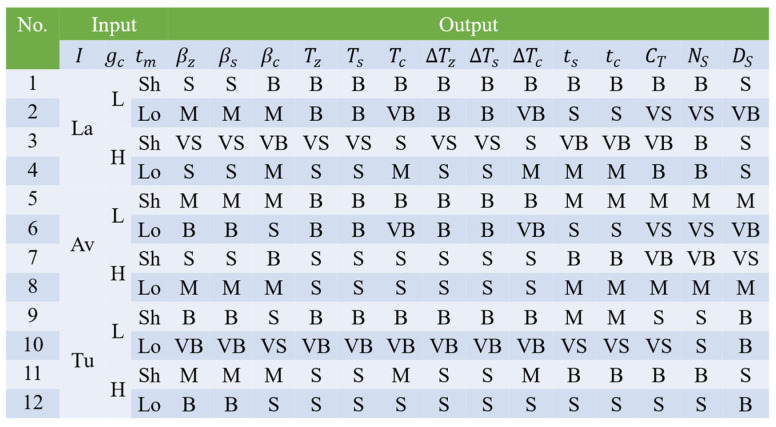
List of fuzzy rules.

Specifically, if the input turbulent intensity I falls into the turbulent set (Tu), the fuzzy outputs increase the turning angle parameters $\beta_{z}$ and $\beta_{s}$; if I belongs to the laminar set (La), these parameters are reduced. Similarly, if the gas concentration $g_{c}$ is classified as high (H), the step length parameters ($T_{z}$, $T_{s}$, $T_{c}$) and their corresponding increments (${∆T}_{z}$, ${∆T}_{s}$, ${∆T}_{c}$) are decreased; if the non-detection time $t_{m}$ falls into the long set (Lo), the maximum execution times $t_{s}$ and $t_{c}$ are shortened. Additionally, when $g_{c}$ is high and $t_{m}$ is short (Sh), the system correspondingly increases $C_{T}$ and $N_{S}$ while reducing $D_{S}$. Furthermore, in situations where the robot has insufficient odor plume and gas concentration information, it is beneficial to increase the parameters $\beta_{c}$, $T_{c}$, and ${∆T}_{c}$ during the cast behavior to rapidly regain plume information. Rather than enumerating every possible interaction, all conceivable input–output combinations are summarized in a table. The system state characterized by I∈La, $g_{c}\in H$, and $t_{m}\in Sh$ is considered the most stable, indicative of maximum information and closest proximity to the odor source, while the state with I∈Tu, $g_{c}\in L$, and $t_{m}\in Lo$ is regarded as the most chaotic, least informative, and furthest from the odor source; all other states are viewed as transitional phases. Based on these qualitative assessments, a set of 12 fuzzy rules has been formulated (as shown in Figure 9) to comprehensively cover all pertinent scenarios, ensuring that the system can make flexible and reasonable decisions even in complex environments.

#### 2.4.5. Defuzzification

Defuzzification is the process by which the fuzzy outputs corresponding to each fuzzy input are converted into a single, precise numerical output. One of the most widely used defuzzification methods is the centroid method, which restores a crisp control signal by calculating the centroid (or center of mass) of the membership function over the numerical domain. The centroid method is defined as

(8) $$x_{out}=\frac{\int x\xi \left(x\right)dx}{\int \xi \left(x\right)dx}$$

In this study, because the required outputs are discrete parameters, we adopt the discrete approximation of the centroid method—namely, the Weighted Average Method—as our defuzzification algorithm. Its formula is given by

(9) $$x_{out}=\frac{\sum_{i=1}^{n}x_{i}\xi \left(x_{i}\right)}{\sum_{i=1}^{n}\xi \left(x_{i}\right)}$$

Among them, *x_out_* represents a definite output parameter value of the fuzzy controller; i is the number of the fuzzy rule (with $i\in \left[1,\;12\right]$); $x_{i}$ represents the central value of the membership function corresponding to the output of the *i*-th rule. In other words, when the *i*-th rule is triggered, the consequent specifies a fuzzy output that can be described by a membership function, and the centroid of this membership function represents the most representative or authoritative output value of the fuzzy set. For instance, the centers of the Gaussian membership function and the Sigmoid membership function are, respectively, determined by the parameter *c* in Formulas (6) and (7), as shown in Figure 8d, where the output *x* corresponds to the five color dots of the fuzzy set. $\xi \left(x_{i}\right)$ represents the membership degree output obtained by calculating the specific values input by the fuzzy controller through the membership function based on the antecedent of the i-th rule. It is also known as the activation degree or connection degree. This value reflects how well the current input state matches the conditions specified by the rule and can range from 0 to 1. $\xi \left(x_{i}\right)$ is close to 1 when the input almost exactly meets the conditions of this rule. Conversely, it tends to zero. In general, in the process of defuzzing, the molecular part represents the sum of the contributions of each rule output $x_{i}$ with their corresponding activation degree $\xi \left(x_{i}\right)$ as the weight adjustment. The denominator part is the sum of all activation degrees, used to weighted average the contributions of these activation degrees, ensuring that the final output $x_{out}$ can reflect the relative contribution ratios of each rule, so that the rules with higher activation degrees occupy a larger proportion in the output, to ensure that the final output is within a reasonable numerical range.

This weighted average method mainly has two advantages. Firstly, it weighted the fuzzy rules, so that those rules with higher matching degree occupied a larger proportion in the final output. Second, it can integrate the fuzzy information provided by multiple triggered rules rather than relying on a single rule, thus generating more balanced and well-considered control decisions. In summary, $x_{i}$ represents the representative value output by the antecedent of each rule, while $\xi \left(x_{i}\right)$ measures the degree to which the antecedent condition of the corresponding rule is satisfied. The two work in synergy through the weighted average method, achieving an effective transformation from fuzzy output to explicit output. This method not only takes into account the balanced distribution of the weights of each rule, but also has high numerical robustness and model interpretability, and thus has been widely applied in practical systems.

## 3. Results and Discussion

### 3.1. Experiment

**Figure 10:**
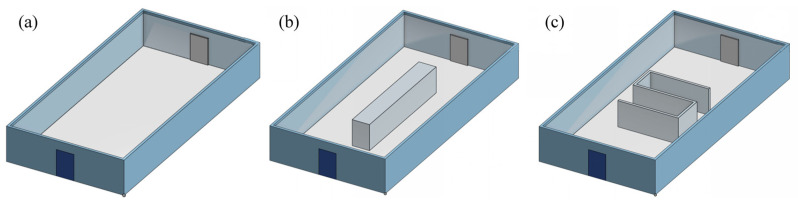
CAD modeling of simulated experimental scenarios (**a**–**c**).

**Figure 11:**
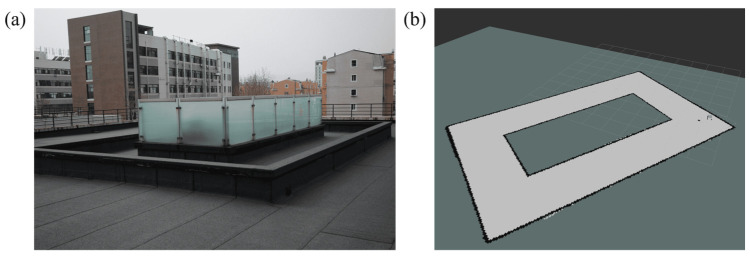
(**a**) Photographs of real experimental scenes; (**b**) Map of the experimental site created using laser SLAM.

This section sets up four sets of experimental scenarios, namely A, B, C and R, representing three simulation experimental sites and one real experimental site, respectively. Among them, A represents an empty room of 20 m × 10 m × 3 m, B represents an obstacle of 10 m × 1.5 m × 2.25 m placed in the center of room A, and C represents an S-shaped obstacle placed in the center of room A. These three simulation environments are first modeled by CAD software (Onshape, PTC, USA, https://www.onshape.com/en/, accessed on 10 December 2025) as shown in Figure 10, and then the fluid simulation inside the site is carried out by CFD software (SimScale GmbH, Munich, Germany; https://www.simscale.com, accessed on 10 December 2025). Then, snapshots are generated by the post-processing software ParaView (version 5.11.1; Kitware Inc., New York, NY, USA). Finally, the GADEN framework under the Robot Operating System (ROS, version 1.16.0; Open Source Robotics Foundation, Mountain View, CA, USA) is used for odor diffusion simulation and robot olfactory navigation experiments. R represents a rectangular platform of 14 m × 7 m × 0.5 m on the roof, with an obstacle covering 10 m × 3 m inside, as shown in Figure 11a is a photo of the site, and Figure 11b is the map created by the ROS robot laser SLAM.

The gas source is generated by the evaporation of anhydrous ethanol liquid. To evaluate the algorithm’s performance under varying airflow conditions, the simulation environment was configured with three average wind speeds: 0.5 m/s, 1 m/s, and 5 m/s. In the actual experiment, despite supplementing the airflow with fans, significant influence from natural wind was unavoidable. This allowed us to test the algorithm’s applicability under real wind field conditions.

**Figure 12:**
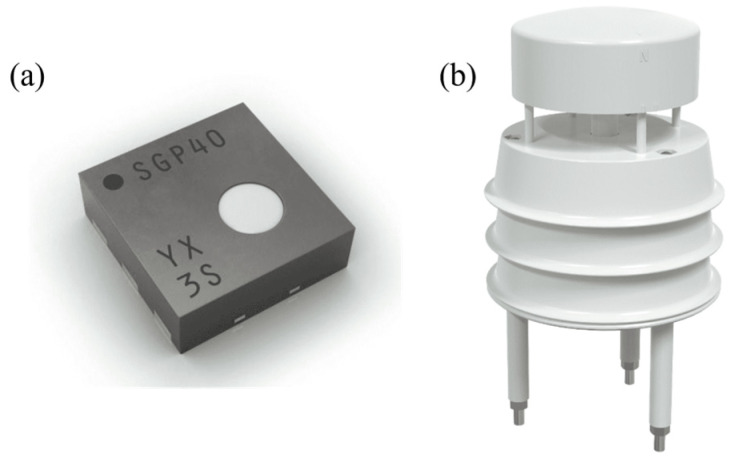
(**a**) Product view of gas sensor; (**b**) Product view of wind speed and direction sensor.

This study selected the SGP40 digital gas sensor, developed by Sensirion (Stäfa, Switzerland), as shown in Figure 12a. The sensor features an ultra-small 6-pin DFN package, with dimensions of only 2.44 × 2.44 × 0.85 mm^3^, and boasts low power consumption, requiring only 2.6 mA current at 3.3 V. This makes it particularly suitable for space-constrained applications, such as integration into robots. The device integrates a complete and user-friendly sensing system on a single chip, equipped with a digital I2C interface and a temperature-controlled micro-hotplate, enabling humidity-compensated indoor VOC air quality detection (e.g., alcohol) with a measurement range of 0–1000 ppm. Its built-in VOC algorithm efficiently converts raw signals into a VOC index, thereby demonstrating excellent performance in terms of environmental adaptability, long-term stability, low drift, and device-to-device consistency. In addition, the sensor exhibits a fast response time of less than 10 s, with data being read every 0.5 s, ensuring timely detection in dynamic environments.

This study also selected a compact ultrasonic wind speed and direction sensor, developed by Vemsee (Shandong Weimengshi Technology Co., Ltd., Jinan, China), as shown in Figure 12b. The sensor uses four ultrasonic probes to cyclically transmit and receive ultrasonic signals in a two-dimensional plane. By detecting changes in ultrasonic transmission time caused by wind, it achieves wind speed and direction measurement without mechanical rotation. The sensor can accurately measure wind speed (range 0–40 m/s, accuracy ±0.5 + 2% FS, resolution 0.01 m/s) and wind direction (range 0–360°, accuracy ±3°, resolution 1°) at any angle, with a response time of only 1 s. It also supports RS485 communication, with a maximum transmission distance of up to 2000 m, and can be easily integrated into robotic systems via an RS485 to USB module.

**Figure 13:**
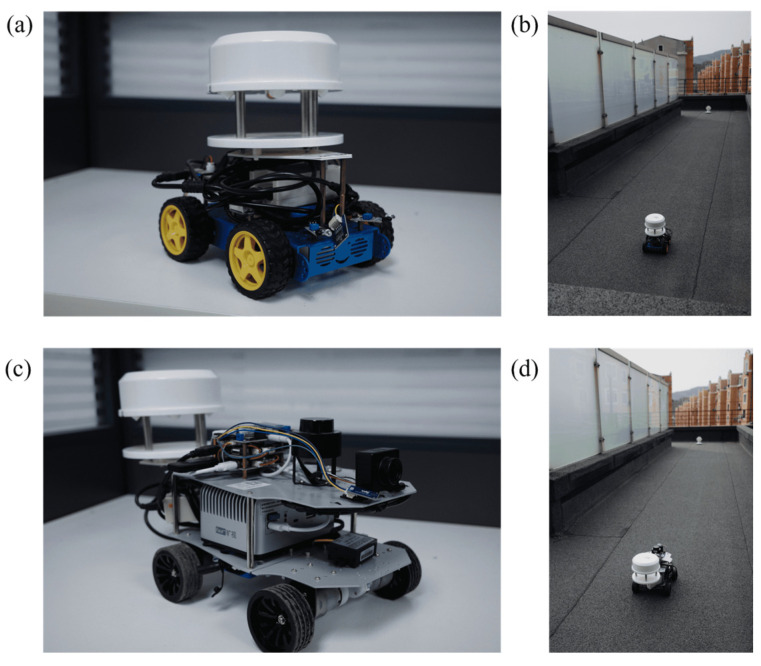
Raspberry Pi robot: (**a**) appearance, (**b**) experimental photos; ROS robot: (**c**) appearance; (**d**) experimental photos.

Finally, this study selected Raspberry Pi robots and ROS robots as experimental platforms, as shown in Figure 13. Both are equipped with the Ubuntu 16.04 operating system. The host computer connects remotely to the robot’s desktop via VNC-Viewer or to the robot’s host via SSH. Due to the limited computational power of Raspberry Pi robots, they are primarily used for verifying simple algorithms like gradient algorithms and bio-inspired algorithms. In contrast, ROS robots, with their stronger processing capabilities, are suitable for running probability algorithms that demand higher computational power.

### 3.2. Results Analysis

**Figure 14:**
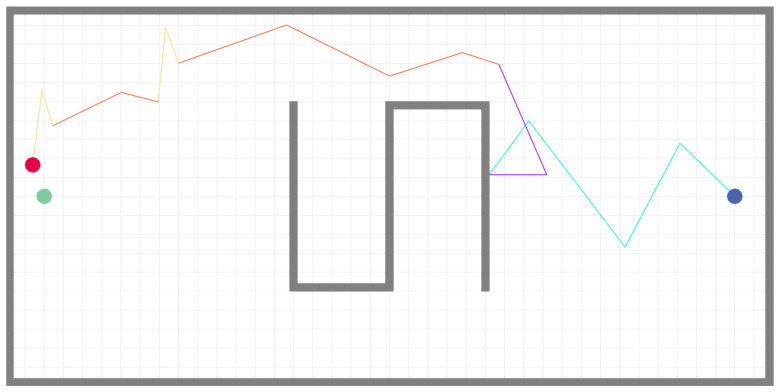
Surge-Cast algorithm with fuzzy controller.

**Figure 15:**
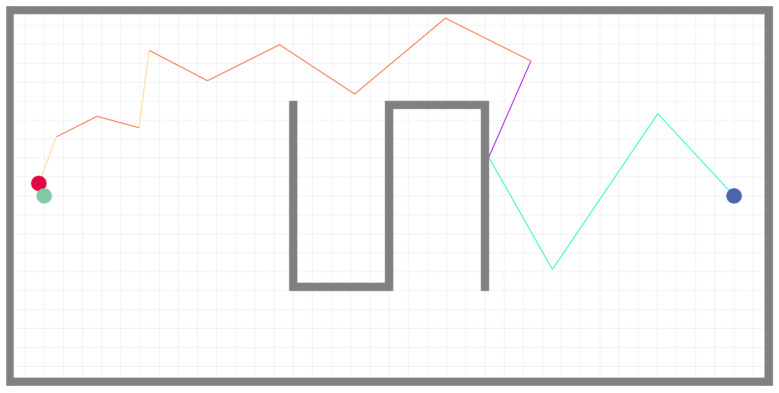
Surge-Cast algorithm without fuzzy controller.

We first present a qualitative case study from the experiments conducted in a simulated environment measuring 20 m × 10 m × 3 m, which featured an S-shaped obstacle at its center. This case is chosen to explicitly link the robot’s movement decisions to the real-time sensory stimuli it encountered. Figure 14 and Figure 15 display the trajectories generated by the robot when executing the surge–cast algorithm before and after the improvements proposed in this chapter.

In these figures, blue dots denote the robot’s starting positions, red dots indicate the termination positions, and green dots mark the odor source. The following analysis will dissect the key decision points in these trajectories (particularly in the improved algorithm of Figure 14), correlating the robot’s actions—such as changes in step length and turning angle—with the concurrent environmental stimuli, including turbulent intensity, gas concentration, and plume loss duration.

To more intuitively demonstrate the role of the fuzzy controller, trajectories corresponding to different search phases are distinguished by different colors. The cyan trajectory represents the Z-shaped search phase; the purple trajectory carries dual meanings—in the original bioinspired algorithm, it depicts the process where the robot takes a step back and then reattempts the current behavior upon encountering an unreachable target, whereas in the improved algorithm it reflects the rebound phase during Z-shaped search when obstacles are encountered; the orange trajectory illustrates the surge behavior; and the yellow trajectory corresponds to the cast behavior.

It is evident from the figures that the fuzzy controller-based surge–cast algorithm enables the robot to make more flexible and adaptive search decisions based on environmental information. Specifically, in the initial region near the starting point—characterized by very low average wind speed, high turbulence intensity, and a considerable distance from both the odor source and the upwind ingress—the available plume information is extremely scarce. Under these conditions, the fuzzy controller prompts the robot to increase its step length, step length increment, and turning angle, thereby broadening its search area to expedite plume detection. After the robot completes the first step of its Z-shaped search, even though the wind speed slightly increases, the gas concentration remains low. Consequently, as the turbulence intensity diminishes, both the turning angles and the step lengths (along with their increments) decrease somewhat; however, as the duration during which the robot fails to detect the plume extends, both the step length and turning angle gradually increase once again. Under these dual influences, the fuzzy controller consistently maintains relatively large step lengths and turning angles to ensure efficient search performance.

Moreover, the rebound-based obstacle avoidance strategy proves to be more efficient than the conventional “backtrack and retry” approach, reducing the overall execution time of the odor localization task in most scenarios. Following this, during the first surge behavior the robot continues to employ large step lengths and turning angles owing to the still-low odor concentration; subsequently, as the odor concentration progressively increases, the robot correspondingly reduces both parameters. Notably, because the robot exits the plume from above at the conclusion of the surge behavior, the ensuing cast behavior is initiated downwards, with the turning angle markedly increasing as the robot draws nearer to the odor source.

This subsection presents a comparative analysis of the experimental results obtained for five algorithms: the Spiral Algorithm, the Surge-Spiral Algorithm, the Surge-Cast Algorithm, the Fuzzy Controller-Based Surge-Spiral (FCSS) Algorithm, and the Fuzzy Controller-Based Surge-Cast (FCSC) Algorithm. The first three algorithms employ a traditional threshold method for odor source declaration, whereas the latter two use a suprathreshold positional aggregation strategy for odor declaration.

**Figure 16:**
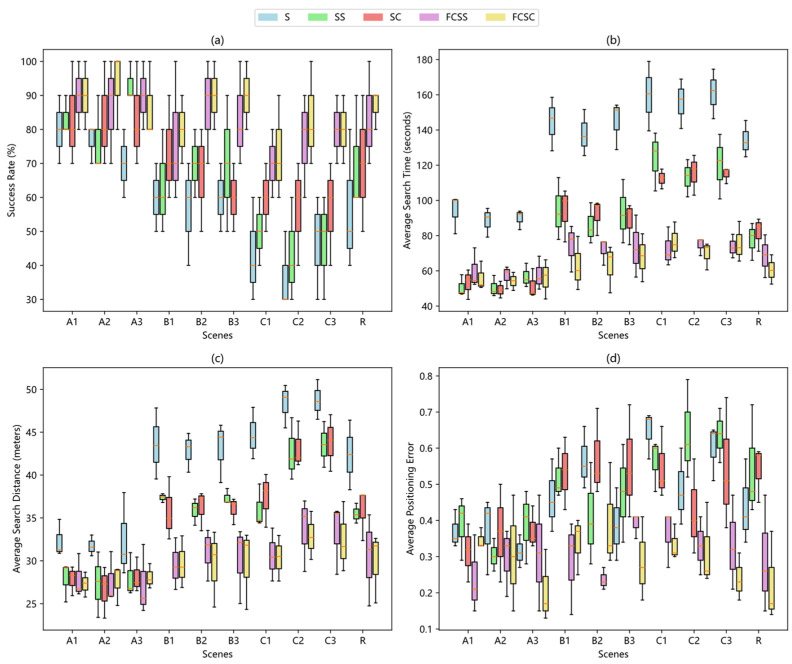
Box plot showing the distribution of experimental results for each algorithm across different scenarios, with an average of 30 experiments per run. (**a**) Comparison of the success rates of different algorithms in different scenarios. (**b**) Average search time of different algorithms in different scenarios. (**c**) Average search distance of different algorithms in different scenarios. (**d**) Average localization error of different algorithms in different environments.

**Figure 17:**
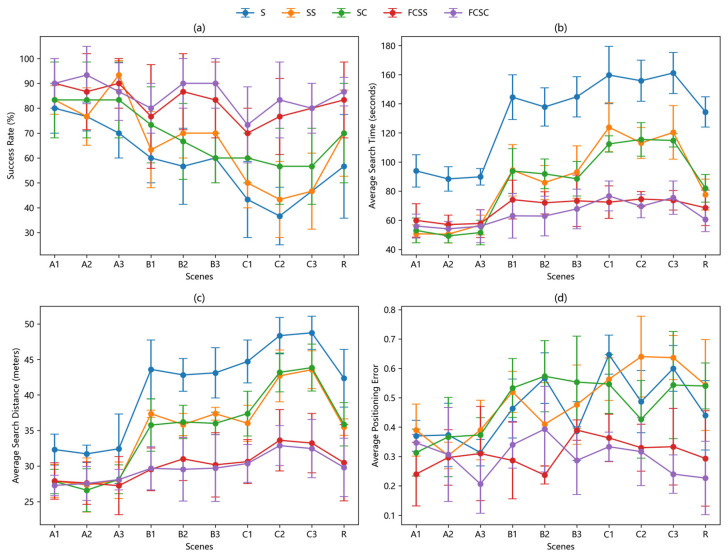
Line Chart showing the distribution of experimental results for each algorithm across different scenarios, complete with standard deviation error bars. (**a**) Comparison of the success rates of different algorithms in different scenarios. (**b**) Average search time of different algorithms in different scenarios. (**c**) Average search distance of different algorithms in different scenarios. (**d**) Average localization error of different algorithms in different environments.

Figure 16 presents the box plots of the experimental results of each algorithm under different experimental scenarios, Figure 17 reflecting the changing trends and algorithmic differences in the four scenario groups. Additionally, to more intuitively demonstrate the overall performance of the five algorithms, the overall average values of the experimental results are provided in Table 1. From the experimental data, it can be seen that the improved moth-inspired algorithm leads in success rate in all test scenarios, and this lead becomes more pronounced in more complex obstacle environments. For instance, the average success rate of the FCSC algorithm in 30 experiments in Scenario A is 6.67% higher than that of the SC algorithm, 20% higher in scenario B, and 21.11% higher in Scenario C. This leading position is mainly attributed to the excellent rebound obstacle avoidance strategy, the plume tracking method based on the lateral improvement of the departure plume, and the adaptive parameter adjustment and behavior switching of the fuzzy controller, which significantly shorten the search time and travel distance. In addition, the air source declaration method that exceeds the threshold position aggregation degree further enhances the positioning accuracy, ensuring that the average positioning error remains stable in both simple and complex scenarios.

It is worth noting that when the experimental scenarios are relatively simple, there is no significant difference in the average search time and search distance between the moth heuristic algorithms before and after improvement. In fact, it is possible that the unimproved algorithm may be more efficient in terms of average search time, such as the SC algorithm in scenario A, which requires 4.07 s less average search time and 0.11 m less average search distance than the FCSC algorithm. This is mainly because in simple scenarios, the increased computational burden of the improved algorithm does not fully leverage its optimization advantages. However, as the environmental complexity increases, the improved algorithm begins to show significant advantages. For example, in scenario C, the FCSC algorithm requires 40.25 s less average search time and 9.57 m less average search distance than the SC algorithm. The average search time and travel distance of the improved algorithm show a steady upward trend, without the sharp increase seen in the unimproved algorithm due to environmental complexity. This clearly demonstrates the strong adaptability of the improved algorithm to complex environments.

Based on the experimental results, it can be analyzed that the spiral algorithm has certain advantages in terms of resource consumption as it does not rely on wind speed and direction information. However, the cost is a significant increase in search time and average search distance. Especially in complex environments, the operation of the spiral algorithm is easily interrupted, which not only severely weakens the algorithm’s execution efficiency but also leads to poor final results. In contrast, the two moth-inspired algorithms can effectively utilize environmental wind information, thereby significantly improving the source-finding efficiency, reducing the search time and distance. However, their source-finding behaviors can also be interrupted, and after the interruption, they only take the strategy of taking one step back and continuing the current behavior, often getting stuck in a repetitive circling situation in front of obstacles and finding it difficult to effectively escape, resulting in time-out and task failure. The two improved moth-inspired algorithms, due to the integration of the rebound strategy, show better performance in escaping obstacles, enabling the robot to avoid obstacles in a more reasonable direction and shorten the search time and distance in most scenarios. At the same time, the plume tracking method based on the lateral improvement of leaving the plume enables the robot to quickly return to the plume with the minimum time and step length, significantly reducing the overall search time and travel distance. It should also be noted that obstacles not only disrupt the behavioral continuity of bio-inspired algorithms but also cause air turbulence, leading to a sharp increase in turbulence intensity and generating a large number of “false sources”, making the robot prone to getting stuck in local optimal states. The adoption of the gas source declaration strategy based on the super-threshold position aggregation degree significantly enhances the robot’s ability to escape from local optimal states and greatly optimizes the positioning result error of the algorithm. Coupled with the advantages of the fuzzy controller in adaptive parameter adjustment and behavior switching, the search time and distance are further shortened, and the overall performance of the algorithm is improved.

### 3.3. Generalization Capability Assessment

**Figure 18:**
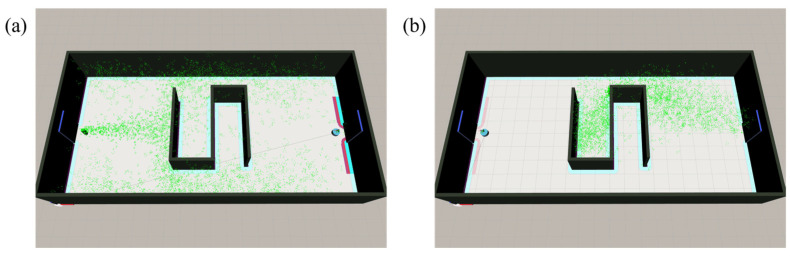
(**a**) Scenario where the robot is located downwind. (**b**) Scenario where the robot is located upwind.

In previous studies, the robot’s initial position was consistently located downwind of the odor source. To evaluate the generalization capability of the algorithm and to simulate more realistic application scenarios, we devised an alternative configuration in which the robot is positioned upwind relative to the odor source. As illustrated in Figure 18, the robot’s location was shifted from (19, 5) to (1, 5), while the odor source was relocated from (1, 5) to (10, 8.25). In this scenario, the trajectory from the robot’s starting point to the point of plume detection is subject to considerable randomness, resulting in greater variability in experimental outcomes under identical conditions.

**Figure 19:**
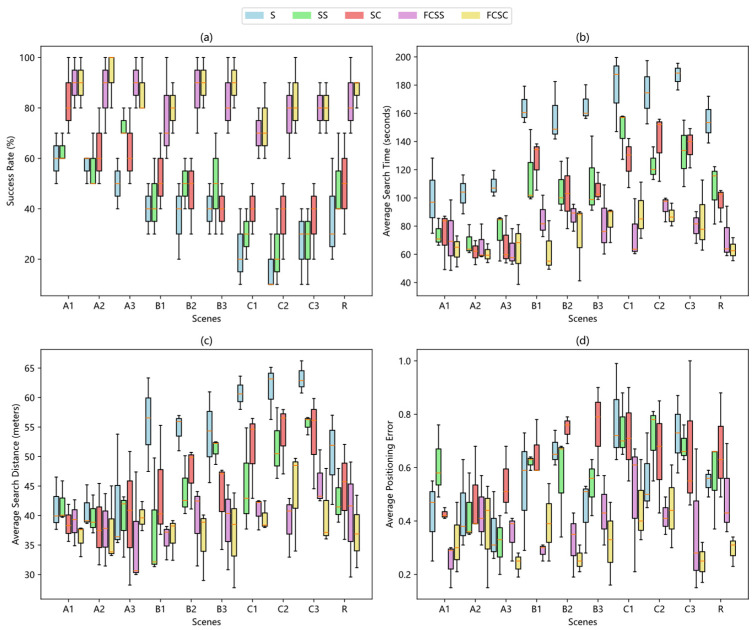
Box plot showing the distribution of experimental results for the robot starting upwind of the gas source, with an average of 30 experiments per run. (**a**) Comparison of the success rates of different algorithms in different scenarios. (**b**) Average search time of different algorithms in different scenarios. (**c**) Average search distance of different algorithms in different scenarios. (**d**) Average localization error of different algorithms in different environments.

**Figure 20:**
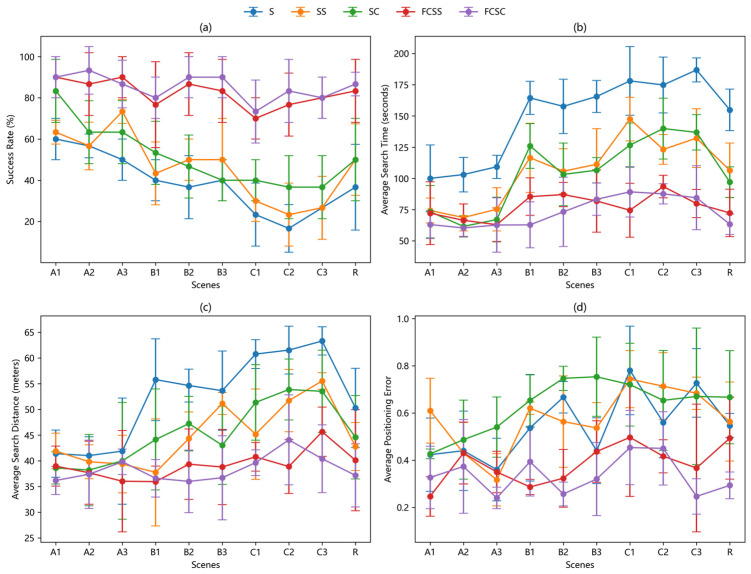
Line graph of the distribution of experimental results where the robot starts upwind of the air source, complete with standard deviation error bars. (**a**) Comparison of the success rates of different algorithms in different scenarios. (**b**) Average search time of different algorithms in different scenarios. (**c**) Average search distance of different algorithms in different scenarios. (**d**) Average localization error of different algorithms in different environments.

According to the comparison of the experimental results shown in Figure 19 and Figure 20, and Table 2, the performance of the traditional bio-inspired algorithm decreases significantly under the condition of the upwind starting point compared with the downwind starting point. This is mainly attributed to the too simple obstacle avoidance strategy of the algorithm, which forces the robot to spend more time and travel distance to get out of the obstacle when it encounters an obstacle, thus affecting the overall performance. In sharp contrast, the experimental results of the two bioinspired algorithms based on fuzzy controller at the upwind start point are worse than those at the downwind start point, but the difference is not significant, showing superior adaptability. This advantage is first attributed to the wide area coverage achieved by adopting a super-step Z-word search strategy guided by fuzzy controller. Especially when the robot is searching against the wind and the odor source is in the opposite direction, the input parameters of the fuzzy controller, gas concentration ($g_{c}$), and the time period ($t_{m}$) during which the robot does not detect the odor plume remain at a low level, enabling the robot to continuously maintain a large step size and turn Angle, thereby quickly getting out of the unfavorable situation and re-capturing the odor plume as soon as possible. In addition, the integrated rebound behavior greatly improves the obstacle avoidance efficiency of the robot and effectively reduces excessive consumption caused by obstacle interference.

## 4. Conclusions

This paper proposes corresponding improvement countermeasures based on the four main deficiencies existing in the traditional moth heuristic algorithm. Firstly, the traditional moth heuristic algorithm does not design an optimization scheme for environments with obstacles, which makes the heuristic behavior of organisms easily interrupted, thereby disrupting the coherence of navigation behavior and significantly reducing the efficiency and success rate of the algorithm. We designed a rebound algorithm, providing a targeted obstacle avoidance strategy for the bio-inspired algorithm. Secondly, in terms of projection behavior, traditional algorithms usually fix the initial projection direction upwards, resulting in inflexibly searching behavior and failing to make full use of historical information. To address this defect, we developed a method based on leaving the plume side to enhance the efficiency of the first projection behavior. Thirdly, traditional algorithms only rely on instantaneous concentration values and preset thresholds to determine the source of odors, and their reliability is obviously insufficient. The gas source declaration method based on the superthreshold location aggregation degree we proposed is a more intelligent and comprehensive determination mechanism. It comprehensively considers environmental factors, sensor characteristics, and the limitations of the algorithm itself to effectively avoid misjudging false odor sources. Fourthly, in terms of the parameter Settings of the bio-inspired algorithm, the surge-projection algorithm needs to pre-set parameters including Z-shaped search, the travel Angle of surge and projection behavior, the initial step size and its increment, the detection threshold of the gas sensor, and the termination threshold for gas source confirmation before the experiment begins. In the traditional surge spiral algorithm, additional parameters such as PI need to be set. Although these parameters can be estimated based on experience, the best parameters obtained in previous experiments are often not fully applicable to the specific context of the current experiment. To determine the optimal performance parameter values suitable for the current experiment, multiple pre-experiments are usually required for repeated trial and error. However, this process not only wastes a great deal of time and energy for researchers but also leads to a significant consumption of energy for the robot and its sensors. Furthermore, whether the parameters are determined by empirical estimation or the pre-experiment method, once the values are fixed, they remain unchanged throughout the search process. However, in the actual environment where the airflow is constantly changing, such a fixed setting is obviously difficult to achieve the optimal source search effect.

Unlike the traditional moth-inspired algorithm that uses fixed parameters to determine the search trajectory of the robot, we have designed an information-based fuzzy controller by deeply exploring the advantages of fuzzy reasoning in dealing with environmental uncertainties and decision-making. This controller adjusts the parameters in real time based on the sensor data currently obtained by the robot, namely gas concentration, wind direction, and wind speed. Therefore, the robot can automatically adjust the search strategy, balance the exploration and utilization during the search process, and adapt to the changes in airflow in the environment. Both simulation and real experiment results show that, compared with two classic olfactory navigation algorithms, the proposed improved algorithm demonstrates higher efficiency and accuracy in terms of success rate, average search time, average search distance, and average positioning error, and performs well under different combinations of odor sources and robot positions.

In conclusion, the above-mentioned method not only effectively supplements the deficiencies of traditional algorithms in theory, but also provides a practical and feasible improvement plan for robot navigation and detection tasks in complex environments. In future work, we will further optimize the fuzzy rules and verify and improve the overall performance of the algorithm in a wider range of application scenarios.

## Figures and Tables

**Table 1:** Overall average of experimental results for each bio-inspired algorithm.

Algorithms	Average Success Rate (%)	Average Search Time (s)	Average Search Distance (m)	Average Positioning Error (m)
S	58.67	131.01	41.02	0.47
SS	66.67	86.64	35.16	0.49
SC	69.33	85.28	35.09	0.48
FCSS	82.33	68.38	30.15	0.31
FCSC	85.33	64.25	29.74	0.30

**Table 2:** Overall average of experimental results for robots starting upwind of the air source.

Algorithms	Average Success Rate (%)	Average Search Time (s)	Average Search Distance (m)	Average Positioning Error (m)
S	38.67	149.43	52.41	0.55
SS	46.67	106.11	44.94	0.58
SC	51.33	103.84	45.45	0.63
FCSS	82.33	77.61	39.21	0.38
FCSC	85.33	72.97	38.42	0.34

## Data Availability

Data are contained within the article.
